# Phytoplankton responses to atmospheric metal deposition in the coastal and open-ocean Sargasso Sea

**DOI:** 10.3389/fmicb.2012.00359

**Published:** 2012-10-12

**Authors:** Katherine R. M. Mackey, Kristen N. Buck, John R. Casey, Abigail Cid, Michael W. Lomas, Yoshiki Sohrin, Adina Paytan

**Affiliations:** ^1^Institute for Marine Science, University of California at Santa CruzSanta Cruz, CA, USA; ^2^Woods Hole Oceanographic InstitutionWoods Hole, MA, USA; ^3^Bay Paul Center for Comparative Molecular Biology and Evolution, Marine Biological LaboratoryWoods Hole, MA, USA; ^4^Bermuda Institute of Ocean Sciences, St George’sBermuda; ^5^University of Hawaii at ManoaHonolulu, HI, USA; ^6^Institute for Chemical Research, Kyoto University, UjiKyoto, Japan

**Keywords:** atmospheric metal deposition, colimitation, copper toxicity, incubation, nutrient addition experiment, picoeukaryote, *Prochlorococcus*, *Synechococcus*

## Abstract

This study investigated the impact of atmospheric metal deposition on natural phytoplankton communities at open-ocean and coastal sites in the Sargasso Sea during the spring bloom. Locally collected aerosols with different metal contents were added to natural phytoplankton assemblages from each site, and changes in nitrate, dissolved metal concentration, and phytoplankton abundance and carbon content were monitored. Addition of aerosol doubled the concentrations of cadmium (Cd), cobalt (Co), copper (Cu), iron (Fe), manganese (Mn), and nickel (Ni) in the incubation water. Over the 3-day experiments, greater drawdown of dissolved metals occurred in the open ocean water, whereas little metal drawdown occurred in the coastal water. Two populations of picoeukaryotic algae and *Synechococcus* grew in response to aerosol additions in both experiments. Particulate organic carbon increased and was most sensitive to changes in picoeukaryote abundance. Phytoplankton community composition differed depending on the chemistry of the aerosol added. Enrichment with aerosol that had higher metal content led to a 10-fold increase in *Synechococcus* abundance in the oceanic experiment but not in the coastal experiment. Enrichment of aerosol-derived Co, Mn, and Ni were particularly enhanced in the oceanic experiment, suggesting the *Synechococcus* population may have been fertilized by these aerosol metals. Cu-binding ligand concentrations were in excess of dissolved Cu in both experiments, and increased with aerosol additions. Bioavailable free hydrated Cu^2+^ concentrations were below toxicity thresholds throughout both experiments. These experiments show (1) atmospheric deposition contributes biologically important metals to seawater, (2) these metals are consumed over time scales commensurate with cell growth, and (3) growth responses can differ between distinct *Synechococcus* or eukaryotic algal populations despite their relatively close geographic proximity and taxonomic similarity.

## INTRODUCTION

The growth of phytoplankton in the ocean is directly influenced by the availability of macro- and micronutrients required for the synthesis of new cells. Compared to micronutrients, macronutrients such as nitrogen (N) and phosphorus (P) are required by cells in higher quantities and at relatively similar ratios across different taxa (Redfield et al., 1963; Bertilsson et al., 2003). In contrast, for trace metal micronutrients, phytoplankton display a broad range of cellular quotas and stoichiometries that can span orders of magnitude (Sunda and Huntsman, 1995b; Bruland et al., 2001). Historically much emphasis has been placed on understanding macronutrient limitation of phytoplankton communities; however, with the development of methods to accurately measure very low metal concentrations in seawater, and improved methods to monitor the growth responses of specific phytoplankton taxa within a mixed population, the effects of trace metals on phytoplankton in the marine environment has become an active field of research (Bruland et al., 1991; Sunda and Huntsman, 1995a,b; Saito et al., 2005; Moore et al., 2006; Paytan et al., 2009).

Atmospheric deposition is a known source of macronutrients to the surface ocean, and the effect of deposition of N (Prospero and Savoie, 1989; Prospero et al., 1996; Paerl, 1997; Bange et al., 2000; Duce et al., 2008; Mackey et al., 2010), Fe (Duce and Tindale, 1991; Mills et al., 2004; Jickells et al., 2005; Moore et al., 2006), and P (Bergametti et al., 1992; Herut et al., 1999; Mackey et al., 2007, 2012) on phytoplankton growth have been documented. While atmospheric deposition is known to supply other biologically important trace metals to the surface ocean, the effect of these trace micronutrients on resident phytoplankton communities remains largely unexplored. A wealth of information is available about the trace metal content of atmospheric aerosols collected at locations around the globe (Duce et al., 1991; Chen et al., 2008; Trapp et al., 2010), and anthropogenic sources appear to have higher fractional metal solubility (and potentially bioavailability) than mineral sources (Sedwick et al., 2007; Sholkovitz et al., 2010, 2012). For some locations, atmospheric deposition may be the main source of certain biologically important metals, such as in the open ocean during seasonal stratification where other potential metal sources like upwelling and discharge from rivers and groundwater are scarce.

There is mounting evidence that the different metal requirements among diverse phytoplankton taxa help determine their distribution, abundance, and activity in the ocean, highlighting a potentially important ecological role for atmospheric metal deposition. For example, addition of Sahara aerosol stimulates N_2_ fixation by diazotrophs in the North Atlantic by providing Fe and P (Mills et al., 2004). Enzymatic requirements for specific metal cofactors can also lead to niche-defining metal requirements for certain groups of phytoplankton. For example, in contrast to eukaryotes, the cyanobacteria *Synechococcus* and *Prochlorococcus* have an absolute cobalt (Co) requirement for which Zn cannot substitute (Sunda and Huntsman, 1995a; Saito et al., 2002), suggesting their distributions in the ocean could be limited by Co availability (Saito and Moffett, 2001; Saito et al., 2005). On the other hand, different toxicity thresholds may influence phytoplankton community composition when metal concentrations are high. Variable toxicity thresholds to copper (Cu) and other trace metals in picophytoplankton has been suggested to influence how phytoplankton community structure responds to atmospheric deposition in the North Atlantic Ocean (Mann et al., 2002), Red Sea (Paytan et al., 2009), and western North Pacific Ocean (Guo et al., 2011). The unique metal requirements and toxicity thresholds of different phytoplankton taxa are therefore important in determining whether segments of the overall population will respond to trace metal availability from atmospheric metal deposition.

The Sargasso Sea is an oligotrophic region in the western North Atlantic Ocean where (co)limitation between macronutrients and trace metals has been identified (Mills et al., 2004; Moore et al., 2008). While atmospheric deposition relieves P and Fe (co)limitation in the Sargasso (Mills et al., 2004), atmospheric N input may have a smaller influence on phytoplankton growth. Michaels et al. (1993) showed that the amount of N contributed by wet deposition is negligible compared to the overall N budget for the region, although it could be important under specific circumstances, such as after large rain events that are followed by calm conditions. Under these conditions, the N-rich rainwater could be retained in the surface ocean for sufficient time to be exploited by phytoplankton before getting diluted through mixing.

Less is known about the effect of dry deposition on phytoplankton in the region and particularly the response of phytoplankton to N and trace metals from this source. The Sargasso Sea receives a mixture of mineral dust from the Sahara Desert and anthropogenic aerosols from North America (Prospero et al., 1996), and receives a moderate amount of dry deposition relative to other open ocean waters (Mahowald et al., 2005). If atmospheric dry deposition provides a similar magnitude of N as wet deposition in the region, as is the case for other areas in the Western Atlantic (Paerl, 1997), then it is likewise doubtful that aerosol N plays a major role in supporting productivity in the Sargasso Sea throughout the year.

In the Sargasso Sea a spring bloom occurs from March to April, when increased irradiance throughout the spring warms surface waters, leading to stratification and trapping relatively high levels of nitrate (${\text{NO}}_{3}^{-}$) from winter mixing above the critical depth. During the bloom, Fe availability affects both the rate of ${\text{NO}}_{3}^{-}$ consumption and the photosynthetic response to changing light regimes (Moore et al., 2006). Rapid phytoplankton growth during the bloom could lead to higher demands for metal micronutrients, and indeed the extent of atmospheric Fe deposition is believed to influence bloom dynamics at some locations in the Sargasso Sea (Moore et al., 2006). However, in light of the knowledge that atmospheric deposition can be an important source of other biologically important metals to remote ocean locations, it seems feasible that deposition of these metals could also shape phytoplankton communities through selective fertilization and toxicity in the Sargasso Sea during the bloom. The greater input of anthropogenic aerosol sources during the spring supports this possibility, because these aerosols generally have higher metal solubility compared to Sahara dust, which dominates during the summer. The responses of phytoplankton to atmospheric metal deposition during the bloom could differ over relatively small spatial scales based on distance from land, depending on the ubiquity and magnitude of terrestrial metal sources such as runoff, groundwater, fluvial discharge, sediment resuspension, mixing, and upwelling.

**FIGURE 1:**
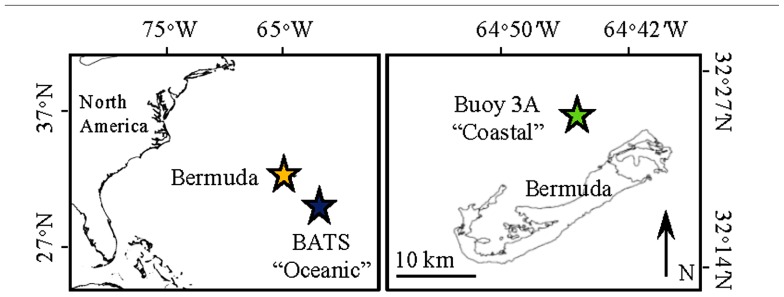
**Map showing the location of the Bermuda Atlantic Time-series Station (BATS) where the oceanic incubation water was collected (blue star) and Buoy 3A where the coastal water was collected (green star).** Orange star in the left panel shows the location of Bermuda.

The goal of this study was to compare the growth responses of phytoplankton in the open-ocean Sargasso Sea and coastal Bermuda (**Figure 1**) to atmospheric deposition during the spring bloom, with an emphasis on the effect of aerosol metal content. To address this goal, we conducted incubation experiments with distinct locally collected aerosols that had different levels of biologically important metals. We monitored changes in phytoplankton growth and trace metal concentration, and show that metals were drawn down more sharply in the open ocean as compared to the coastal site. Moreover, phytoplankton subpopulations responded differently to aerosol additions between the two sites, likely due to different trace metal requirements and metal speciation. We explore the responses of phytoplankton to metal availability in the coastal and open, and discuss how atmospheric deposition may influence biogeochemical cycles by providing trace metals to metal limited phytoplankton communities.

## MATERIALS AND METHODS

### AEROSOL COLLECTION AND ANALYSIS

Weekly integrated (over ~7 day periods) atmospheric particulate samples were collected at the Tudor Hill observatory in southwestern tip of Bermuda on top a 15 m tall sampling tower as described in (Arimoto et al., 1995; Sholkovitz and Sedwick, 2006). Air was pumped and aerosols collected for all wind directions except when the wind traversed Bermuda directly [contingent on wind speed (>1 m/s) and direction (210–315°)], thereby eliminating potentially contaminating air masses from local emissions on the island. Aerosol samples were collected on acid cleaned quartz filters and stored frozen until analysis. The soluble metal content of each aerosol sample was determined following dissolution as described in Buck et al. (2006). A 100 mL volume of Milli-Q water was passed using vacuum filtration through a 47 mm diameter subsection of the sample filter, and the filtrate was acidified to (pH 2) using ultrapure trace metal grade nitric acid (Optima). Metal concentrations in the filtrate were measured on an Element 2 inductively coupled plasma-mass spectrometer (ICP-MS). Scandium and Rhodium were added to each sample for internal standardization. Mixed trace metal standards were prepared over a range of concentrations (1–100 ppb) from concentrated certified trace metal stock solutions diluted in 2% nitric acid. The mass of total suspended particles (TSP) on the filter was calculated from the aluminum (Al) content, assuming 10% solubility and an Al:TSP ratio of 0.101, which is typical for Bermuda aerosols (Prospero et al., 1996).

### INCUBATION EXPERIMENTS

Nutrient and aerosol addition incubation experiments were conducted in April 2010 with surface water collected at open ocean (“oceanic”) and coastal locations in the Sargasso Sea (**Figure 1**). The oceanic experiment used water collected at the Bermuda Atlantic Time-series Station (BATS; 31°40′N, 64°10′W). The coastal experiment used water collected at Buoy 3A (32°24.531′N, 64°44.769′W) located within the Bermuda platform, inside of the Rim Reef Zone that encircles the waters north of the island. In 2010, deep mixing to 300–400 m occurred at BATS during February, and stratification began to occur in March. By April at the time of our sampling the mixed layer depth had shoaled to ~80 m, though stratification was punctuated by periods of deeper mixing down to ~120 m in the weeks leading up to our sampling. For both experiments, water was collected on windy days reaching 4–6 on the Beaufort scale.

Water was transported back to the Bermuda Institute of Ocean Science (BIOS), where both experiments were conducted. Water was dispensed into acid cleaned, sample rinsed 500 mL clear polycarbonate incubation bottles, and treatments were made as described below (12 bottles per treatment). Bottles were incubated under shading material (50% light attenuation) in a tank with circulating seawater to maintain surface ocean temperature. Three bottles from each treatment were collected at each time point, including time zero (t0), which was processed immediately and completed within 1 h after the nutrient or aerosol treatments were added, as well as after 1, 2, and 3 days of incubation. Samples for monitoring phytoplankton growth and nutrient and metal analysis were collected as described below.

**FIGURE 2:**
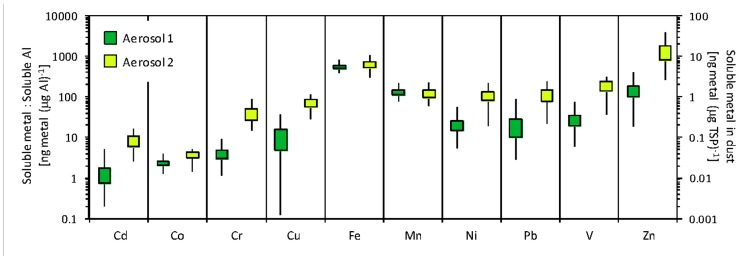
**Freshwater-dissolved trace metals in the two types of aerosol used in the incubation experiments**. Primary *y*-axis shows the ratio of dissolved metal to dissolved Al, which tends to be higher for anthropogenic aerosols. Secondary *y*-axis shows the amount of freshwater dissolvable metal per mass of aerosol. The mean is at the center of each box, the upper and lower edges of the box depict ± SE, and lines show range. Of the 15 samples extracted, nine were classified as Aerosol type 1 and six were classified as Aerosol type 2. The individual aerosol samples used in the incubation experiment were selected from among these samples, and their metal contents are given in **Table 1**.

In each experiment, treatments included inorganic nutrient additions, aerosol additions, and controls (no nutrient or aerosol additions). Inorganic nutrient treatments included single addition or a combination of 0.2µM ${\text{PO}}_{4}^{3-}$ , 10µM ${\text{NO}}_{3}^{-}$ , and 5 nM Fe. The following inorganic nutrient treatments were included: ${\text{PO}}_{4}^{3-}$ alone, ${\text{NO}}_{3}^{-}$ alone, Fe alone, and ${\text{PO}}_{4}^{3-}$ , ${\text{NO}}_{3}^{-}$ , and Fe together (hereafter referred to as the “N,P,Fe” treatment). For the aerosol treatments, we selected two aerosol samples (Aerosol 1 and Aerosol 2) based on the chemical composition of the dissolved fraction in each sample as previously determined in the lab (see **Figure 2**; **Table 1**). Each type of aerosol was added at two concentrations, for a total of four aerosol treatments. The higher deposition treatments (referred to as “Aerosol 1” and Aerosol 2” for simplicity) simulated a concentration of aerosol that would occur in the surface ocean upper 10 m mixed layer following 10 days of deposition after a moderately strong deposition event typical of the central North Atlantic (20 g m^-2^ y^-1^). Lower aerosol concentration treatments were also tested, simulating a typical annual average deposition rate for the Sargasso Sea near Bermuda (2 g m^-2^ y^-1^; Mahowald et al., 2005), and these treatments are referred to as “Aerosol 1 low” and “Aerosol 2 low” to differentiate them from the high treatments.

**Table 1:** Freshwater-dissolved trace metal content of the aerosol samples used in the incubation experiment.

Metal	Aerosol 1 (ng metal/μg TSP)	Aerosol 2 (ng metal/μg TSP)
Cd	0.051	0.123
Co	0.026	0.051
Cr	0.094	0.508
Cu	0.361	1.057
Fe	8.100	6.711
Mn	1.786	1.864
Ni	0.571	1.745
Pb	0.885	1.505
V	0.737	2.766
Zn	4.075	10.95

### PHYTOPLANKTON GROWTH RESPONSES

Aliquots (1.5 mL) for flow cytometry were preserved with 75 µL 10% paraformaldehyde solution, incubated in the dark at room temperature for 10 min, and frozen at -80°C until analysis. Samples were analyzed on an Influx flow cytometer (BD Biosciences, Franklin Lakes, NJ, USA) triggering on forward angle light scatter (FSC). Cell abundances and particulate organic carbon (POC) contribution were determined for each group (**Figures 4A–H**). Populations of *Synechococcus* and eukaryotic algae were identified in all samples and discriminated based on their characteristic fluorescence and scattering properties (**Figure 4I**). Two subpopulations of eukaryotic algae lacking orange/yellow (580/20 nm bandpass) autofluorescence were also detected in both coastal and oceanic samples, and were discriminated by FSC-H amplitude (eukaryotic algae 3A and 3B, herein). *Prochlorococcus* was found only in very low abundances in coastal waters, and was a minor community member in terms of cell abundance and POC contribution; accordingly, we focus our discussion on the eukaryote and *Synechococcus* subpopulations. The low *Prochlorococcus* abundances are not likely due to the low autofluorescence of these cells being below detection limits of the flow cytometer, since the same protocol and flow cytometer used to enumerate these cells for routine monitoring at BATS was employed in this study. Possible explanations for the low *Prochlorococcus* abundances are explored in the discussion. Quality flagging during batch processing was defined to reject gated populations which failed the following criteria: events <100, kurtosis <0, skewness >|5| , median absolute deviation >50%. The cellular carbon (C) content of *Synechococcus* and picoeukaryote cells during each experiment was determined based on forward scatter signal following Casey et al. (2012). The contribution of these cells to particulate organic C was determined by multiplying cellular C content by cell abundance.

For chlorophyll *a* (chl *a*) analysis, 250 mL seawater was filtered through GFF filters (Whatman). Filters were frozen at -20°C until analysis. Chl *a* concentration was determined fluorometrically following 24 h 90% acetone extraction at -20°C. Fluorescence was measured on an AU10 fluorometer (Turner Designs) calibrated with chl *a* standard solution derived from *Anacystis nidulans* cyanobacteria following (JGOFS, 1994).

### SEAWATER NITRATE AND TRACE METAL ANALYSES

Seawater samples for nutrient analysis were collected by syringe filtration (0.45µM PES) and stored frozen until analysis. ${\text{NO}}_{3}^{-}$ concentrations were measured on a flow injection autoanalyzer (FIA, Lachat Instruments Model QuickChem 8000) using standards prepared in Milli-Q water. Blanks were prepared in aged, low nutrient seawater. The detection limit based on three-times the standard deviation of the blanks was determined to be 0.1µM for ${\text{NO}}_{3}^{-}$ . ${\text{PO}}_{4}^{3-}$ was measured as described above for ${\text{NO}}_{3}^{-}$ , with a detection limit of ~0.01µMol L^-1^, and is discussed in Mackey et al. (2012). We report here only ${\text{NO}}_{3}^{-}$ (including trace amounts of nitrite and ammonium) data as growth in the ${\text{PO}}_{4}^{3-}$ treatment was not appreciably different than controls in any of the experiments.

To measure trace metals dissolved in seawater, 50 mL subsamples were collected by syringe filtration (0.2µM PES) in a laminar flow hood. For simplicity we only report data for Aerosol 2 “high” in comparison to the control and N,P,Fe treatments, as phytoplankton growth indicated that trace metal limitation may have been relieved by this treatment (see below). Samples were acidified with four drops of ultrapure trace metal grade hydrochloric acid (Optima) to pH < 2. Trace metals were separated from the major seawater ions and pre-concentrated by a factor of ~3 using NOBIAS Chelate PA-1, a non-swelling resin with ethylenediaminetriacetic acid functional groups (Sohrin et al., 2008; Sohrin and Bruland, 2011). Metal concentrations in the eluant were determined on an Elan DRC II ICP-MS. Metal drawdown was quantified by calculating the difference between mean concentrations at t0 and t3, and uncertainty in the difference was calculated by propagating the error according to the standard error propagation formula: (A ± a) – (B ± b) = (A - B) ± √(a^2^ + b^2^).

Statistical significance was evaluated at *p* < 0.05 and *p* < 0.10 performing a one-way analysis of variance (ANOVA) to detect differences between the mean values, followed by Dunnett’s test to compare control values with other treatments for each experiment.

### COPPER SPECIATION MEASUREMENTS

Copper speciation measurements were performed at two analytical windows in the control, Aerosol 1, and Aerosol 2 treatments in the coastal and oceanic experiments. Samples were collected at t0, as well as t3. To obtain a sufficient volume of sample, 200 mL of sample water was combined from each of the three replicate bottles at each time point. The composite sample was passed through an in-line cartridge filter (0.45µM, Osmonics, PTFE membrane) using a peristaltic pump, and stored frozen (-20°C) until analysis.

Copper speciation was measured using competitive ligand exchange- adsorptive cathodic stripping voltammetry (CLE-ACSV) using the added ligand salicylaldoxime (SA) as described previously (Buck and Bruland, 2005; Buck et al., 2010). Briefly, thawed speciation samples were buffered with 7.5 mM ammonium-borate buffer (final pH 8.2, NBS scale), titrated with 0–15 nM of dissolved Cu and allowed to equilibrate for at least 2 h before the addition of SA as the competing ligand. Following an additional equilibration time of 15 min, each vial in the titration was analyzed by ACSV. Two analytical windows were employed in analyses using SA additions of 2.5µM and 0.5µM. Titration data were interpreted using van den Berg/Ružic (Ružic, 1982; van den Berg, 1982) and Scatchard (Scatchard, 1949) linearization techniques, and the results from both linearizations were averaged to provide final ligand concentrations and conditional stability constants, with standard deviations of these values reflecting the variability between interpretation method output for the titrations (Buck et al., 2010). One ligand class was determined within each analytical window with the ligand determined in the higher analytical window denoted “stronger” while the lower analytical window defined the “weaker” ligand class. Free, hydrated Cu^2+^ concentrations were determined using the higher analytical window data, which better represents the ambient speciation, following the calculations presented by Moffett and Dupont (2007).

## RESULTS

### AEROSOLS CHEMISTRY

To identify unique aerosol “types” to be used in the incubation experiment, the concentration of dissolved trace metals in Milli-Q water was measured in aerosol samples collected in Bermuda. Of the 15 samples analyzed, two qualitatively defined types of aerosol were identified based on the relative amounts of dissolved trace metals in the sample (Aerosol type 1 *n* = 9; and Aerosol type 2 *n* = 6). The concentration ranges for each metal in these aerosol samples are shown in **Figure 2**. We selected individual filters from each of these types to use as the aerosol additions in the incubation experiments. Aerosol 1 was collected over November 2–9, 2009 and Aerosol 2 was collected over September 28, 2009 to October 12, 2009 and October 26, 2009 to November 2, 2009. Aerosol 2 had relatively higher amounts of dissolved Cd, Cr, Cu, nickel (Ni), Pb, V, and Zn than Aerosol 1, while dissolved Fe and manganese (Mn) concentrations were similar between the two types (**Table 1**).**Aerosol 2 had twice as much dissolved Co (0.051 ng Co (µg TSP)^-1^) as Aerosol 1 (0.026 ng Co (µg TSP)^-1^).

Vanadium (V) enrichment relative to Al has been used to identify anthropogenic influence in aerosol samples, because anthropogenic aerosols are enriched in V while the main source of Al in aerosols is from the earth’s crust (Chen and Duce, 1983). In this study, the ratio of dissolved V to dissolved Al was higher in Aerosol type 2 (192 ± 49 ng V (µg Al)^-1^) than Aerosol type 1 (27 ± 7 ng V (µg Al)^-1^), which indicates a higher fraction of anthropogenic material in Aerosol type 2. The higher content of other dissolved trace metals in Aerosol type 2 (**Figure 2**) is also consistent with greater anthropogenic influence.

### PHYTOPLANKTON GROWTH

**FIGURE 3:**
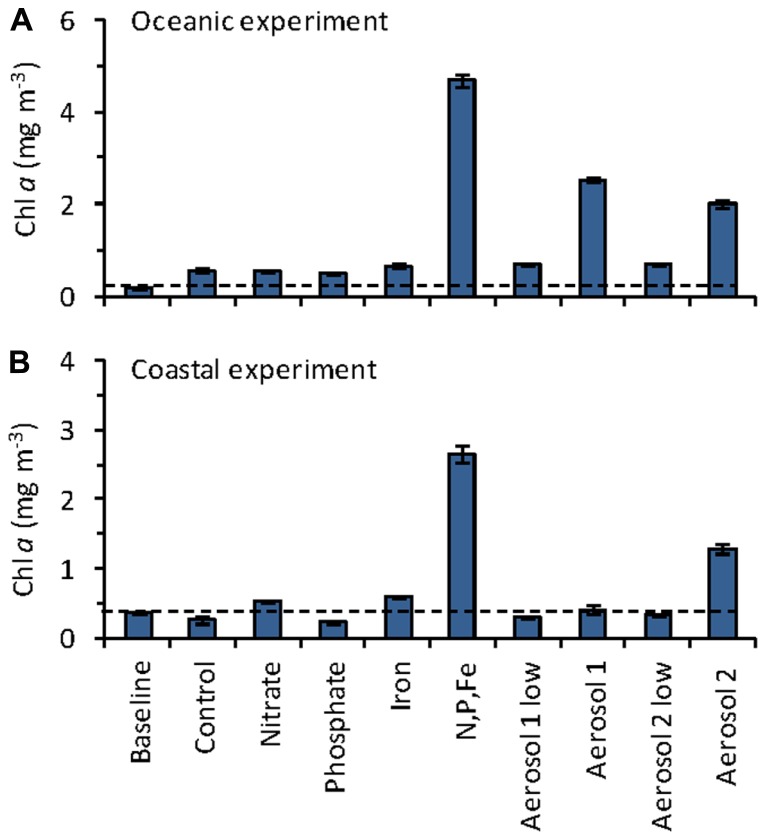
**Mean chlorophyll *a* concentrations on day 3 of the (A) oceanic, and **(B)** coastal incubation experiments**. Dashed line shows the initial (baseline) chl *a* level. Error bars show standard error for triplicate bottles.

The initial chl *a* concentration in the oceanic experiment was 0.17 mg m^-3^. The greatest increases in chl *a* after 3 days of incubation were observed in the N,P,Fe, Aerosol 2, and Aerosol 1 treatments, reaching 4.7, 2.5, and 2.0 mg m^-3^, respectively (**Figure 3A**). All other treatments showed levels close to the control (range 0.50–0.69 mg m^-3^ in day 3). In the coastal experiment, the initial chl *a* concentration was 0.35 mg m^-3^, and the largest increases were observed for the N,P,Fe and Aerosol 2 treatments, which reached 2.7 and 1.3 mg m^-3^, respectively (**Figure 3B**). All other treatments had levels close to the control (range 0.21–0.59 mg m^-3^). Because substantial growth responses were only observed in the “N,P,Fe” and both the “Aerosol 1 high” and “Aerosol 2 high” treatments, the remainder of our discussion focuses on these treatments, along with the controls for each experiment. For simplicity, we refer to these treatments as “Aerosol 1” and “Aerosol 2” as described above.

**FIGURE 4:**
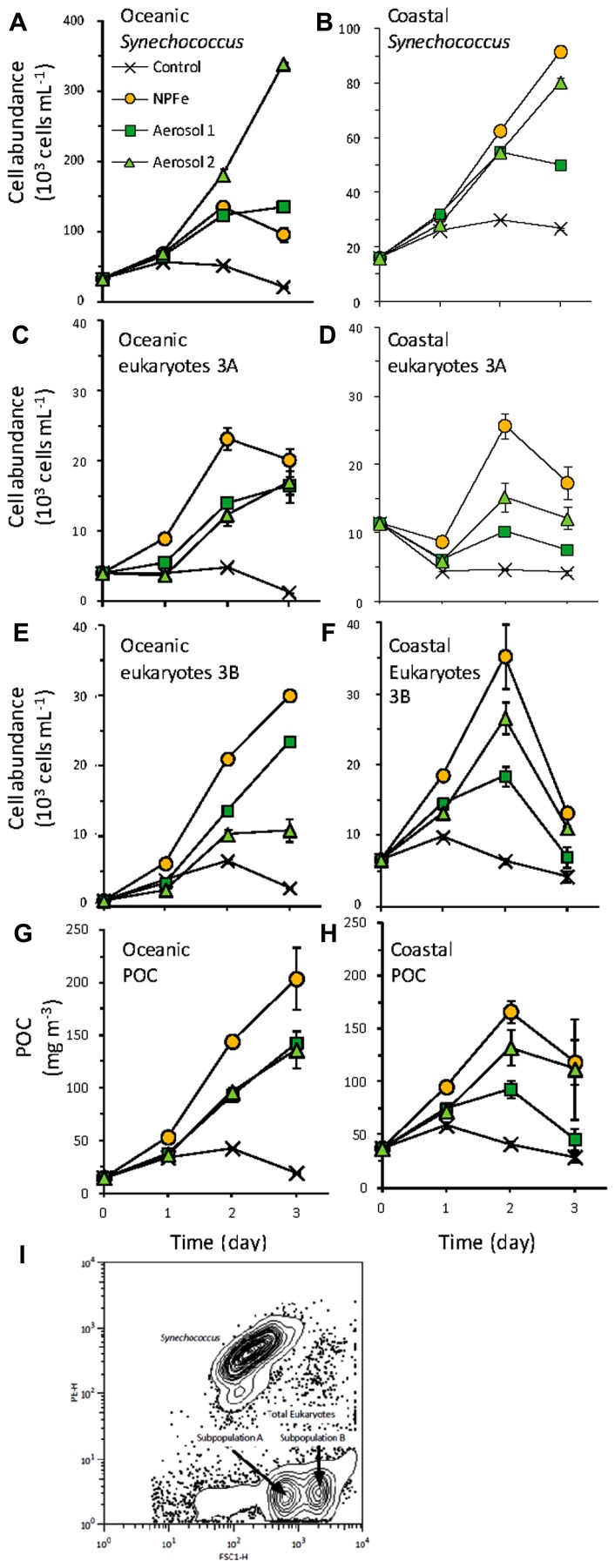
**Cell growth for (A,B) *Synechococcus***(C,D)** picoeukaryote subpopulation 3A, and **(E,F)** picoeukaryote subpopulation 3A during the oceanic **(A,C,E)** and coastal **(B,D,F)** experiments. **(G,H)**** Total particulate C concentration contributed from *Synechococcus* and picoeukaryotes in the **(E)** oceanic, and **(F)** coastal experiments. Error bars show standard error for triplicate bottles. **(I)** Distributions of *Synechococcus* and picoeukaryote subpopulations based on forward scatter (FSC-H) and orange fluorescence (PE-H).

Initial cell abundances, cellular carbon content and fluorescence were statistically indistinguishable between treatments for each population and subpopulation reported (*p *> 0.05). In the oceanic experiment, the initial phytoplankton popula- tion was dominated by *Synechococcus* (31.7 ± 0.5 × 10^3^ cells mL^-1^; **Figure 4A**), followed by picoeukaryotes (8.6 ± 0.2 × 10^3^ cells mL^-1^; **Figures 4C,E**). *Synechococcus* abundance increased 10-fold in the Aerosol 2 treatment (reaching 339.2 ± 9.6 × 10^3^ cells mL^-1^), and approximately fourfold in the Aerosol 1 (135.5 ± 7.5 × 10^3^ cells mL^-1^) and N,P,Fe treatments (96.0 ± 1.1 × 10^3^ cells mL^-1^). Two subpopulations of eukaryotes, 3A and 3B, were initially present at 5.6 ± 0.2 and 1.2 ± 0.1 × 10^3^ cells mL^-1^, respectively. Total picoeukaryote growth of both subpopulations responded most strongly in the N,P,Fe treatment, increasing sixfold (51.9 ± 1.3 × 10^3^ cells mL^-1^). The Aerosol 1 and Aerosol 2 treatments also resulted in increased total picoeukaryote growth, reaching 39.1 ± 0.2 × 10^3^ and 41.2 ± 4.0 × 10^3^ cells mL^-1^, respectively. Eukaryotic subpopulations 3A and 3B reached the highest concentrations at t3 in the N,P,Fe treatment (20.1 ± 1.6 and 30.0 ± 0.7 × 10^3^ cells mL^-1^, respectively). While subpopulation 3A responded identically between Aerosol 1 and Aerosol 2 treatments (16.5 ± 1.2 and 17.0 ± 2.9 × 10^3^ cells mL^-1^, respectively; *p* = 0.88), subpopulation 3B concentrations were significantly higher in Aerosol 1 (23.5 ± 0.8 × 10^3^ cells mL^-1^) than Aerosol 2 (10.8 ± 1.6 × 10^3^ cells mL^-1^; *p* = 0.006).

The initial cellular C content in the oceanic experiment for *Synechococcus* was 223 ± 4 fg C cell^-1^. Both Aerosol 1 and Aerosol 2 treatments resulted in decreased carbon quotas at the end of the incubations (205 ± 7.5 and 181 ± 6.6 fg C cell^-1^, respectively) while N,P,Fe treatment resulted in the highest cellular quotas (250 ± 1.1 fg C cell^-1^). Carbon quotas for eukaryotic subpopulations 3A and 3B increased significantly throughout all incubation treatments (initially 829 ± 3.5 and 2953 ± 11 fg C cell^-1^). Subpopulation 3A increased similarly, in the control, Aerosol 2, and N,P,Fe treatments to 1019 ± 4.0 fg C cell^-1^ at t3, each significantly higher than Aerosol 1 (963 ± 5.2 fg C cell^-1^; *p *< 0.05). Subpopulation 3B also increased significantly but less than subpopulation 3A for all treatments (2809 ± 61 fg C cell^-1^) compared to the control 3140 ± 27 fg C cell^-1^; *p *< 0.05).

The initial amount of POC contributed by *Synechococcus* and picoeukaryotes was 15.3 ± 0.6 mg C m^-3^, and by the end of the experiment the control remained close to this level (16.7 ± 0.5 mg C m^-3^; **Figure 4G**). Aerosol 1 and Aerosol 2 treatments caused similar increases in POC, reaching 143 ± 4.4 mg C m^-3^ and 136 ± 17.8 mg C m^-3^ by t3, respectively. The greatest increase was observed in the N,P,Fe treatment, where POC increased to 204 ± 29.3 mg C m^-3^. As reflected in cell abundance data, the highest biomass increases for *Synechococcus* were 10-fold for Aerosol 2 treatment. Although total eukaryotic algae biomass increases were again highest (15-fold) for the N,P,Fe treatment, subpopulation responses differed considerably; subpopulation 3A increased fourfold while subpopulation 3B increased 23-fold.

In the coastal experiment *Synechococcus* and picoeukaryotes were present at more similar abundances (17.8 ± 0.7 and 18.0 ± 0.4 × 10^3^ cells mL^-1^) at the start of the experiment (**Figure 4**). Over 3 days, *Synechococcus* numbers increased fivefold in the Aerosol 2 and N,P,Fe treatments (80.3 ± 2.1 and 91.4 ± 4.4 × 10^3^ cells mL^-1^), and threefold in the Aerosol 1 treatment (49.9 ± 0.9 × 10^3^ cells mL^-1^). Picoeukaryote abundances increase for the first 2 days of incubation but then decreased during the final day for all treatments. Total picoeukaryotes also responded most strongly in the Aerosol 2 and N,P,Fe treatments, reaching 41.8 ± 3.1 × 10^3^ and 60.9 ± 4.8 × 10^3^ cells mL^-1^, respectively. Total picoeukaryotes in the Aerosol 1 treatment reached only 28.6 ± 1.5 × 10^3^ cells mL^-1^ by the last day of the experiment.

The initial cellular C content in the coastal experiment was 211 ± 1 fg C cell^-1^ for *Synechococcus*. Carbon quotas at the end of the experiment were statistically indistinguishable between control and both Aerosol treatments (209 ± 6 fg C cell^-1^; *p *> 0.05). Carbon quotas increased to 257 ± 6 fg C cell^-1^ by t3 in the N,P,Fe treatment, significantly higher than other treatments (*p *< 0.001). The initial picoeukaryote cellular C content was 953 ± 14 fg C cell^-1^ for subpopulation 3A and 3177 ± 25 fg C cell^-1^ for subpopulation 3B. In all treatments carbon quotas decreased throughout the incubation to 885 ± 10 fg C cell^-1^ for subpopulation 3A and 2787 ± 83 fg C cell^-1^ for subpopulation 3B at t3.

The initial POC contributed by *Synechococcus* and picoeukaryotes in the coastal experiment was 47.5 ± 0.5 mg C m^-3^ (**Figure 4H**). Autotrophic biomass declined slightly in the control by the end of the experiment (30.5 ± 3.7 mg C m^-3^). For the treatments, biomass increased for the first 2 days of incubation (maximum for N,P,Fe treatment reached 167 ± 10.3 mg C m^-3^) and then declined in the last day of the experiment at t3, reaching final POC concentrations of 45.7 ± 10.3 mg C m^-3^, 112 ± 48.0 mg C m^-3^, and 119 ± 21.1 mg C m^-3^ for Aerosol 1, Aerosol 2, and N,P,Fe treatments, respectively. At t3 subpopulation 3A biomass was significantly higher in the N,P,Fe experiment (*p *= 0.043).

### NITRATE AND TRACE METAL DRAWDOWN

**FIGURE 5:**
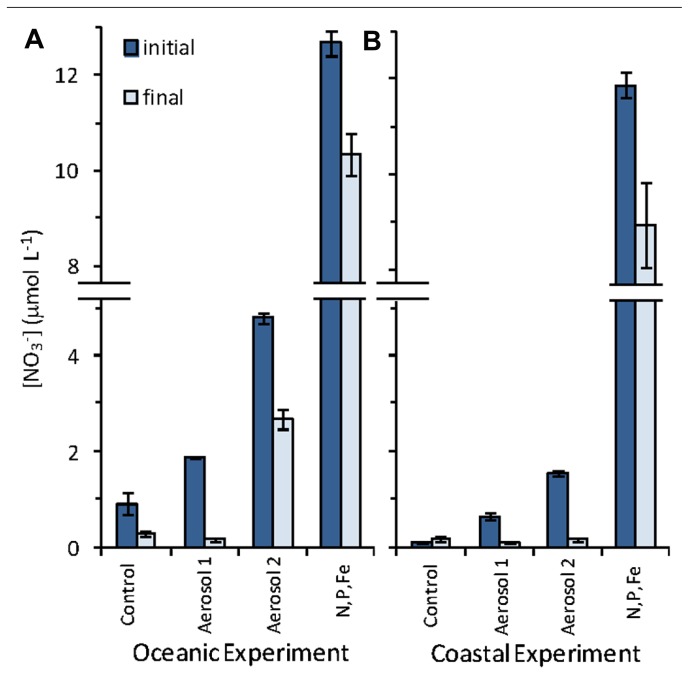
**Initial and final ${\text{NO}}_{3}^{-}$ concentrations in the **(A)** oceanic experiment, and **(B)** coastal experiment**. Error bars show standard error for triplicate bottles.

The ambient ${\text{NO}}_{3}^{-}$ concentrations in the untreated control bottles were 0.91 ± 0.23µM in the oceanic experiment and 0.11 ± 0.02µM in the coastal experiment (**Figure 5**). Addition of aerosol to the incubation water increased the concentration of ${\text{NO}}_{3}^{-}$ above background levels. In the oceanic experiment, the ${\text{NO}}_{3}^{-}$ concentration almost doubled increasing to 1.9 ± 0.23µM in the Aerosol 1 treatment and increased fivefold to 4.8 ± 0.11µM in the Aerosol 2 treatment. About 2µM ${\text{NO}}_{3}^{-}$ was drawn down by the final time point for both treatments (**Figure 5**). In the coastal experiment ${\text{NO}}_{3}^{-}$ was enriched to 0.63 ± 0.07µM in the Aerosol 1 treatment and to 1.5 ± 0.07µM in the Aerosol 2 treatment. The lower apparent enrichment in the coastal samples could be due to rapid ${\text{NO}}_{3}^{-}$ uptake during the 1 h it took to process the samples after aerosol additions were made. By the end of the coastal experiment, nearly threefold more ${\text{NO}}_{3}^{-}$ was drawn down in the Aerosol 2 treatment (1.4µM) as compared to the Aerosol 1 treatment (0.55µM).

**FIGURE 6:**
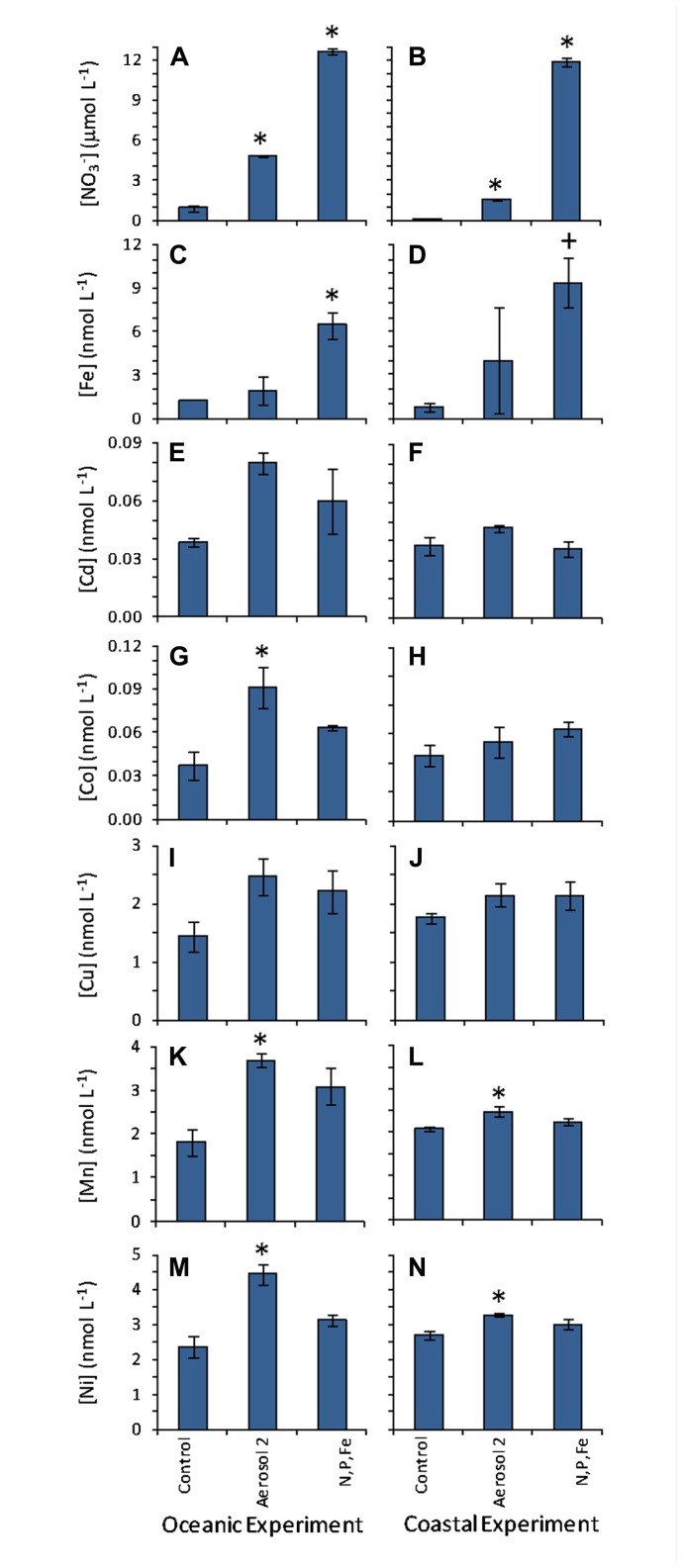
**Initial ${\text{NO}}_{3}^{-}$ and seawater-dissolved metal concentrations measured at t0**. Left-hand column shows values in the oceanic experiment and right-hand column shows values in the coastal experiment for **(A,B)** ${\text{NO}}_{3}^{-}$ ; **(C,D)** Fe; **(E,F)** Cd; **(G,H)** Co; **(I,J)** Cu; **(K,L)** Mn; **(M,N)** Ni. ${\text{NO}}_{3}^{-}$ data is re-plotted from **Figure5** for easier comparison with the trace metal data. Error bars show standard error. Levels that were statistically different from the initial control incubation water at time zero are indicated by “*” for *p* < 0.05, and “+” for *p* < 0.10.

Background trace metal concentrations in the coastal and oceanic incubation water were within the range of concentrations typically observed in the open ocean (**Table 2**; Sohrin and Bruland, 2011; Jickells and Burton, 1988; Sedwick et al., 2005). Aerosol 2 had higher dissolved metal content that Aerosol 1 (**Table 1**; **Figure 2**). Because the Aerosol 2 treatment induced the strongest growth response in *Synechococcus* in the oceanic experiment (**Figure 4A**) despite showing similar ${\text{NO}}_{3}^{-}$ drawdown as Aerosol 1 (**Figure 5A**), we measured the concentrations of seawater-soluble trace metals only in the Aerosol 2 incubation water (both open ocean and coastal) and compared that to the levels in the control and the N,P,Fe treatment (which also induced growth) with the goal of identifying which metal(s) might have been responsible for this growth response. Addition of Aerosol 2 doubled the concentrations of Fe, Cd, Co, Cu, Mn, and Ni above background levels at t0 (**Figure 6**). These concentrations however, are still within the typical range for open ocean waters (**Table 2**). Enrichment of metals other than Fe was also evident in the N,P,Fe treatment at t0; these were likely introduced from trace amounts of these metals in the salts used to prepare the nutrient amendments.

**Table 2:** Trace metal concentrations in seawater used in the oceanic and coastal incubation experiments before any nutrient or aerosol additions were made.

Metal	Background concentration in oceanic experiment water (mean ± SE)	Background concentration in coastal experiment water (mean ± SE)	Estimated mean concentration in open ocean seawater*	Concentration range in open ocean seawater*
Cd	0.04 ± 0.002	0.04 ± 0.005	0.6	0.001–1.05
Co	0.04 ± 0.010	0.05 ± 0.007	0.04	0.003–0.3
Cu	1.4 ± 0.26	1.8 ± 0.084	3	0.4–5
Fe	1.3 ± 0.047	0.73 ± 0.30	0.5	0.03–3
Mn	1.8 ± 0.31	2.1 ± 0.055	0.3	0.06–10
Ni	2.4 ± 0.30	2.7 ± 0.12	8	2–12

*The concentrations were within the range of typical values for open ocean water (Sohrin and Bruland, 2011; ranges include surface and deep waters). Units are nmol L^-1^*

* *Values from Sohrin and Bruland (2011)*.

**FIGURE 7:**
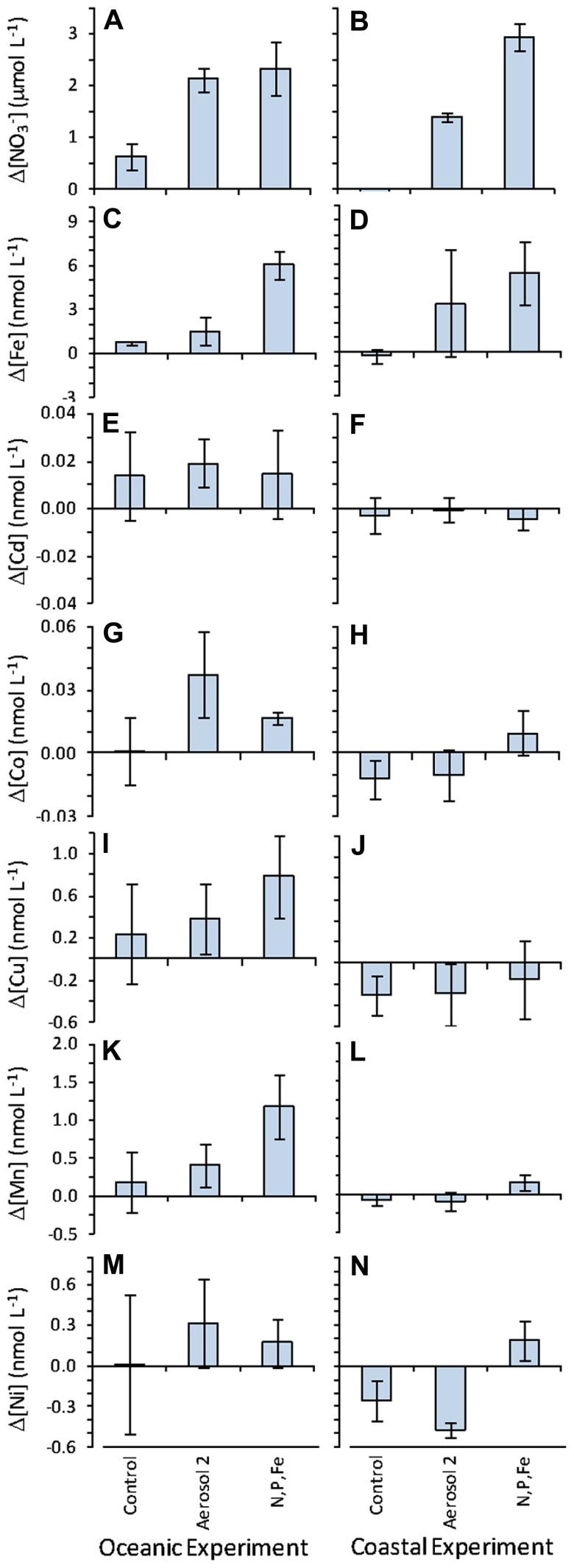
**Change in seawater-dissolved ${\text{NO}}_{3}^{-}$ and metal concentrations over 72 h measured as the difference between values at t0 and t3**. Positive values indicate the nutrient was drawn down over the course of the experiment, and values close to zero indicate little change over the course of the experiment. Left-hand column shows values in the oceanic experiment and right-hand column shows values in the coastal experiment for **(A,B)** ${\text{NO}}_{3}^{-}$ ; **(C,D)** Fe; **(E,F)** Cd; **(G,H)** Co; **(I,J)** Cu; **(K,L)** Mn; **(M,N)** Ni. Error bars show standard error for the difference between mean concentrations at t0 and t3.

Drawdown of dissolved trace metals during the experiment was determined by calculating the difference between t0 and t3 concentrations, and propagation of error was done to determine standard errors of the differences. Drawdown of Fe, Cd, Co, Cu, Mn, and Ni was apparent in the oceanic experiment in the Aerosol 2 and N,P,Fe treatments (**Figure 7**). The largest variability was seen in dissolved Fe content for bottles treated with Aerosol 2 in the coastal experiment (**Figure 6D**), and is typical of an incubation study using non-homogenized natural aerosol samples due to natural variability in the size and composition of the aerosol particles as distributed on the filter (Cahill and Wakabayashi, 1993; Mackey et al., 2010). In contrast to the oceanic experiment, there was less change over time in the concentration of dissolved metals in the coastal experiment (**Figure 7**), indicating little net drawdown of these metals during the incubation. In a few instances (e.g., for Cu and Ni) final concentrations were higher than initial concentrations, and could be the result of low biological demand for these metals together with continuous release of metals from the added aerosols over time, as has been observed for aerosol ${\text{PO}}_{4}^{3-}$ (Ridame and Guieu, 2002; Mackey et al., 2012).

### COPPER SPECIATION

**FIGURE 8:**
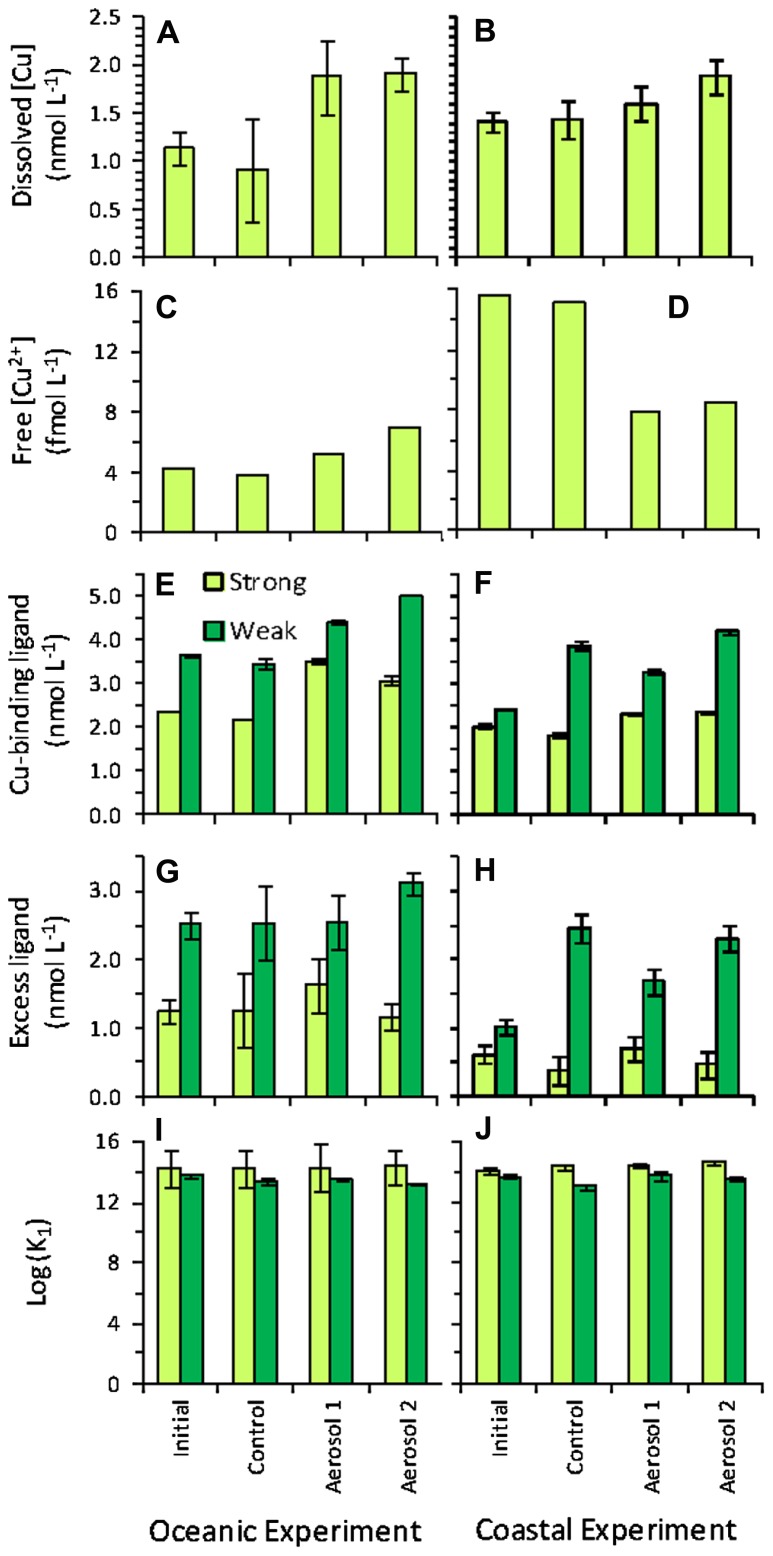
**Copper chemistry for initial (baseline) samples, and final (t3) samples for control, Aerosol 1, and Aerosol 2**. Left-hand column shows values in the oceanic experiment and right-hand column shows values in the coastal experiment for **(A,B)** dissolved Cu concentration; **(C,D)** free Cu^2+^ concentration; **(E,F)** Cu-binding ligand concentration; **(G,H)** excess ligand concentration; **(I,J)** log K.

The initial (t0) soluble Cu concentration in the oceanic experiment was 1.14 ± 0.18 nM, and final concentrations at t3 were similar to initial levels in the control (0.92 ± 0.53), and enriched for both Aerosol 1 and 2 (~1.9 nM; **Figure 8A**). In the coastal experiment, the initial Cu concentration was 1.41 ± 0.10 nM. The final Cu concentration in the control also did not change significantly by t3 (1.43 ± 0.19 nM), whereas enrichment occurred in Aerosol 1 (1.60 ± 0.18 nM) and Aerosol 2 (1.88 ± 0.18 nM; **Figure 8B**).

Two ligand classes were identified in the experiments, a stronger ligand class (average log $K_{{\text{CuL}}_{2},\;{\text{Cu}}^{2+}}^{\text{cond}}=14.38\,\;\pm 0.17,\;n=16)$ in the higher analytical window and a weaker ligand class (average log $K_{{\text{CuL}}_{2},\;{\text{Cu}}^{2+}}^{\text{cond}}=13.52\,\;\pm 0.29,\;n=16)$ in the lower analytical window. Both ligand classes were present in concentrations in excess of dissolved copper in all samples. Excess stronger Cu-binding ligands ([L_1_]–[Cu]) increased in the Aerosol 1 addition above initial and control samples in both the oceanic and coastal experiments, while excess L_1_ in the Aerosol 2 treatments were similar to t3 control concentrations in both experiments (**Figure 8**). Excess L_1_ concentrations were similar between initial and t3 control bottles in the oceanic experiment, but decreased between initial and t3 control samples in the coastal experiment. Excess weaker Cu-binding ligands ([L_2_]–[Cu]) increased in the Aerosol 2 treatment for the open ocean experiments, but were similar or lower than the control in the coastal experiment. Excess L_2_ concentrations were similar between t0 initial, t3 controls and the Aerosol 1 treatment in the open ocean experiment. In the coastal experiment, excess L_2_ concentrations more than doubled between the initial t0 and the control t3, Aerosol 1 and Aerosol 2 bottles (**Figure 8**).

Bioavailable free, hydrated Cu^2+^ (hereafter referred to simply as free Cu^2+^) concentrations were low (<10^-13^ M) and well below toxicity thresholds for phytoplankton in all samples. Free Cu^2+^ levels differed markedly between the oceanic and coastal experiments (**Figures 8C,D**). In the oceanic experiment (**Figure 8C**), the initial free Cu^2+^ concentration and the final concentration in the control were both ~4 fM. Aerosol 1 caused a slight enrichment in Cu^2+^ (~5 fM). The highest free Cu^2+^ was observed in the Aerosol 2 treatment, where Cu^2+^ nearly doubled relative to initial levels (~7 fM). In contrast, the amount of free Cu^2+^ decreased in aerosol treatments in the coastal experiment (**Figure 8D**). Initial and final control levels were high (~16 fM), and decreased by half in both Aerosol 1 and 2 (~8 fM).

## DISCUSSION

### PHYTOPLANKTON COMMUNITY DYNAMICS

The potential for atmospheric deposition to alter phytoplankton community composition via selective fertilization (Mills et al., 2004; Moore et al., 2006) and toxicity (Mann et al., 2002; Paytan et al., 2009) of different taxonomic groups has been demonstrated in various ocean waters. In the present study, we sought to evaluate the range of responses that coastal and open ocean phytoplankton communities in the Sargasso Sea would have following simulated atmospheric deposition events during the spring bloom period. Based on chl *a* levels, the greatest increases in autotrophic biomass occurred in the N,P,Fe and the Aerosol 1 and 2 treatments. Drawdown of ${\text{NO}}_{3}^{-}$ occurred in both oceanic and coastal experiments for Aerosol 1 and Aerosol 2 (**Figure 5**). In contrast, ${\text{PO}}_{4}^{3-}$ levels for Aerosol 1 and Aerosol 2 stayed the same in the coastal experiment (not shown), and actually increased in the oceanic experiment due to gradual aerosol P dissolution from the aerosols and DOP remineralization over the course of the experiment (Mackey et al., 2012). Chl *a* levels remained similar to the control at t3 following treatment with N alone, suggesting that autotrophic biomass at the coastal and oceanic sites required Fe and possibly additional metals to show maximal growth at the time of our experiments.

We compared the growth of different members of the phytoplankton community to determine differences in taxon-specific responses to atmospheric deposition and how this varied between sites, or within the same site for aerosols with different chemical compositions. At the time of our experiments the phytoplankton community at both locations was dominated by picoeukaryotes and *Synechococcus*. The absence of oceanic *Prochlorococcus* is notable, as it is typically the numerically dominant phytoplankter at the BATS site throughout much of the year. *Prochlorococcus* population crashes are observed annually following the maximal thermal convective mixing depth event (DuRand et al., 2001), depending on the phase and amplitude of the North Atlantic Oscillation index (NAOi) and associated regional weather (Hurrell and Deser, 2009). A particularly cold winter and strong negative anomaly of the NAOi was perhaps responsible for late winter 2010 mixing depths to exceed 400 m (not shown), leading to enhanced *Prochlorococcus* population crashes preceding our sampling.

Picoeukaryotes at both oceanic and coastal locations responded most strongly to the N,P,Fe addition (**Figure 4**), consistent with chl *a* responses. Aerosol 1 and Aerosol 2 both induced moderate increases in picoeukaryote abundances in the oceanic experiment (**Figures 4C,E**), whereas Aerosol 2 induced more picoeukaryote growth than Aerosol 1 in the coastal experiment (**Figures 4D,F**). Important differences in the response of picoeukaryote subpopulations to aerosol treatments were observed in both oceanic and coastal experiments. For instance, initial subpopulation 3B abundances were lower than for subpopulation 3A, but the rapid growth of subpopulation 3B allowed it to out-compete subpopulation 3A by the end of the oceanic experiment, such that their abundances were more comparable. This shift in the dominance of picoeukaryote subpopulations suggests that atmospheric deposition induces competition among picoeukaryotes causing changes in population distribution and abundance. Additionally, atmospheric deposition influenced the carbon dynamics within this group. Picoeukaryotes, as identified through flow cytometry, are an operationally defined group, so the increase in abundance of the larger, subpopulation 3B cells in the oceanic experiment tended to increase the overall cellular POC content of picoeukaryotes relative to initial values.

Coastal *Synechococcus* abundance also responded differently to different treatments. Like the overall picoeukaryote community, *Synechococcus* abundance increased more in response to Aerosol 2 (fivefold increase) than Aerosol 1 (threefold increase), reaching levels comparable to the N,P,Fe treatment (**Figure 4B**). The stronger growth responses of picoeukaryotes and coastal *Synechococcus* to Aerosol 2 than Aerosol 1 could stem from the greater content and drawdown of ${\text{NO}}_{3}^{-}$ in the coastal experiment for Aerosol 2 (1.9µM added and ~1.5µM drawdown) compared to Aerosol 1 (0.6µM added and ~0.5µM drawdown, **Figure 5B**). Treatment with N alone, P alone, or Fe alone did not stimulate growth (**Figure 3B**), whereas treatment with N,P,Fe induced strong growth responses, although no P drawdown was observed in these samples (not shown). Together these results suggest that oceanic and coastal picoeukaryotes and coastal* Synechococcus* growth responded strongly to N and Fe, and that atmospheric aerosols were able to relieve this limitation.

The growth response of oceanic *Synechococcus* to aerosol additions differed markedly from coastal *Synechococcus* populations (**Figures 4A** and B). Unlike the coastal population, oceanic *Synechococcus* did not respond as strongly to the inorganic nutrient addition of N,P,Fe, in keeping with the observation that oceanic *Synechococcus* respond more favorably to smaller (nanomolar) ${\text{NO}}_{3}^{-}$ additions (Glover et al., 2007). The largest oceanic *Synechococcus* growth response occurred when Aerosol 2 was added, which resulted in a nearly 10-fold increase in *Synechococcus* abundance. Unlike the coastal experiment, the drawdown of ${\text{NO}}_{3}^{-}$ was similar in the Aerosol 1, Aerosol 2, and N,P,Fe treatments in the oceanic experiment (~2µM drawdown, **Figure 5**), suggesting that the stronger growth of *Synechococcus* was probably not due to differences in ${\text{NO}}_{3}^{-}$ uptake. Likewise, the greater growth response in Aerosol 2 was likely not due to Fe since Aerosol 1 and Aerosol 2 contributed similar dissolved Fe. Rather, our results indicate that the growth may have been due to relief from limitation by a trace metal micronutrient, since Aerosol 2 had a higher compliment of most dissolved metals than Aerosol 1 (**Table 1**; **Figure 2**).

To determine which micronutrients in Aerosol 2 might have contributed to the strong growth response of *Synechococcus* in the oceanic experiment, we compared the initial levels and calculated the drawdown of several biologically important metals to see which had been drawn down as the *Synechococcus* bloomed (**Figure 7**). In the oceanic experiment, drawdown of Fe, Cd, Co, Cu, Mn, and Ni all occurred following addition of Aerosol 2. Additionally, initial levels of Co, Mn, and Ni were significantly higher in the aerosol 2 treatment compared to the control (**Figure 6**), whereas they were not significantly higher than the control in the N,P,Fe treatment. This suggests that aerosol-derived Co, Mn, or Ni may have contributed to the strong *Synechococcus* growth response to Aerosol 2.

Unlike eukaryotic phytoplankton, *Synechococcus* has an absolute requirement for Co that cannot be satisfied by other metals. Eukaryotic phytoplankton, including diatoms and coccolithophores, are able to substitute Zn for Co, thereby maintaining growth even when Co levels are vanishingly low (Sunda and Huntsman, 1995a; Saito and Goepfert, 2008). In contrast, *Synechococcus* cells cannot substitute Zn for Co, and their populations have been shown to correlate with dissolved Co concentrations in the Sargasso Sea (Saito and Moffett, 2001) and the Costa Rica Upwelling Dome in the Equatorial Pacific Ocean (Saito et al., 2005). Moreover, concentrations of Co-binding ligands also co-vary with *Synechococcus* cell abundances at these locations, leaving open the possibility that *Synechococcus* produces these organic chelating compounds to help acquire Co where Co concentrations are in the low picomolar range. The high affinity of *Synechococcus* for Co is one of many adaptations that could contribute to high *Synechococcus* abundance during the spring bloom in the Sargasso Sea. The preference of *Synechococcus* to utilize ${\text{NO}}_{3}^{-}$ as a N source may also promote *Synechococcus* growth over that of *Prochlorococcus*, which tends to dominate this region during the stratified season when ${\text{NO}}_{3}^{-}$ levels are very low (Moore et al., 2008; Martiny et al., 2009).

Relief from Co limitation is a possible explanation for the strong *Synechococcus* growth response observed in response to the Aerosol 2 addition. However, this explanation does not rule out the possibility that *Synechococcus* were driven to Co limitation during the experiment by the high levels of ${\text{NO}}_{3}^{-}$ in Aerosol 2, or that they benefited from other metals that were introduced by Aerosol 2. Additionally, it is possible that other phytoplankton and bacteria may have contributed to the drawdown of Co we observed in the Aerosol 2 treatment. The cellular Co quota for cultured *Synechococcus bacillaris* ranges from 0.08 to 1.43µMolCo molC^-1^, similar to the Co content of particulate material in the Sargasso Sea in which picoplankton dominate (~1.5µMolCo molC^-1^; Sherrell and Boyle, 1992). In the oceanic experiment, *Synechococcus* POC increased ~70 mgC m^-3^ in response to the Aerosol 2 treatment (**Figure 4G**). Taking the higher cellular Co quota estimate of 1.5µMolCo molC^-1^, this increase in *Synechococcus* POC would have only required ~9 pM Co uptake, suggesting that picoeukaryotes and other microbes may have also contributed to the 40 ± 20 pM Co drawdown we observed.

Other biologically relevant metals, such as Mn and Ni, were also drawn down over the course of the experiment, suggesting that they too fueled the growth of the phytoplankton community. Mn is an important plant micronutrient, and is a central component of the oxygen evolving complex of photosystem II in all photosynthetic organisms, including phytoplankton (Falkowski and Raven, 2007). Mn is also a cofactor in the superoxide dismutase (SOD) enzyme of diatoms that protects cells from oxidative stress (Wolfe-Simon et al., 2006). Similarly, Ni is a cofactor in some (cyano)bacterial SOD enzymes (Palenik et al., 2003), as well as a cofactor for the urease enzymes of many marine phytoplankton (Price and Morel, 1991; Palinska et al., 2000). The sharp drawdown of these metals during the oceanic incubation experiment suggests that aerosol-associated Mn and Ni enrichment has the potential to supply phytoplankton with these vital micronutrients in the open ocean.

In addition to requiring metals for nutritional purposes, the higher resistance to Cu toxicity in *Synechococcus* compared to *Prochlorococcus* has also been proposed to shape phytoplankton community structure in the Sargasso Sea, particularly during the summer-stratified period (Mann et al., 2002). The bioavailability of dissolved Cu has been shown to be a function of free Cu^2+^ concentrations, and not total dissolved Cu concentrations (Sunda and Guillard, 1975). As dissolved Cu is typically complexed by strong organic ligands in seawater, Cu bioavailability is dependent upon ambient Cu speciation. In our oceanic experiment, Cu^2+^ concentrations were similar between the initial and t3 control bottles, increased slightly in the Aerosol 1 treatment, and nearly doubled over control concentrations in the Aerosol 2 treatment. In contrast, in the coastal experiment, Cu^2+^ concentrations in the initial and t3 control were roughly fourfold higher than observed in the initial and control bottles of the oceanic experiment, and decreased by nearly half in both the Aerosol 1 and Aerosol 2 treatments.

Cu^2+^ concentrations in the oceanic and coastal experiments were well below toxicity thresholds for marine phytoplankton, including *Synechococcus* species (toxicity threshold ~10^-11^ M; Brand et al., 1986) in all samples and treatments. Thus, Cu toxicity is not likely to have had an effect on phytoplankton growth in these experiments. This is consistent with previous Cu addition experiments in the Sargasso Sea showing no decrease in the growth of *Synechococcus* following 2 nM Cu additions (Mann et al., 2002).

The changes in Cu^2+^ concentrations in the oceanic and coastal experiments reflect the changes in total dissolved Cu and strong Cu-binding ligands measured in the experiments. In the absence of a change in L_1_ concentrations, the increase in dissolved Cu measured from the aerosol treatments would have increased Cu^2+^ concentrations in both the coastal and oceanic experiments. However, only in the Aerosol 2 treatment of coastal waters, where dissolved Cu concentrations (1.88 nM) would have exceeded the L_1_ concentrations measured in the t3 control (1.82 nM), would Cu^2+^ concentrations have increased dramatically and approached toxicity thresholds for *Synechococcus* in the absence of Cu-binding ligand production. *Synechococcus *have been shown to produce Cu-binding ligands under Cu toxicity stress in the laboratory (Moffett and Brand, 1996), but there is no indication of Cu toxicity in any of the treatments and *Synechococcus *growth was much higher in the Aerosol 2 treatment than Aerosol 1. It is not obvious, then, why excess Cu-binding ligands in both experiments were highest in the Aerosol 1 treatments, and similar between initial, t3 control, and t3 Aerosol 2 treatments.

It is also notable that initial Cu-binding ligand concentrations were in greater excess of dissolved Cu in the oceanic waters than in the coastal waters. This is counterintuitive if Cu-binding ligands are primarily produced by cyanobacteria to alleviate Cu toxicity, as Cu^2+^ concentrations were well below toxicity thresholds throughout both of these experiments, even after aerosol-associated Cu additions. The sources and function of Cu-binding ligands, particularly in oceanic waters, merits further attention, as the conventional wisdom linking Cu toxicity with ligand production does not fit well here. Recent studies have highlighted the role of Cu as a micronutrient in the ocean, limiting large diatom growth under low Fe conditions (Peers et al., 2005; Peers and Price, 2006). However, experiments in this study were dominated by picoeukaryotes and cyanobacteria under relatively Fe replete conditions, hence regulation of nutritional Cu uptake may be more complicated than previously believed.

*Prochlorococcus* was not abundant in our incubation water, so we were unable to directly assess how aerosol-derived Cu would affect this population. The results of Mann et al. (2002) show that Cu toxicity to *Prochlorococcus* in this region can occur following 2 nM or less Cu enrichment. Our results show that following moderate amounts of aerosol enrichment, Cu^2+^ levels remained well below the toxicity threshold for *Prochlorococcus *during the spring. However, the summer-stratified period generally has more large atmospheric deposition events, and Cu-binding ligand concentrations are ~twofold lower than during winter mixing (Moffett, 1995; Mann et al., 2002). This leaves open the possibility that aerosol-Cu enrichment could potentially curb *Prochlorococcus* growth in the summer-stratified period, but this would likely be limited to exceptionally large deposition events of aerosols with high fractions of soluble Cu.

### THE IMPACTS OF ATMOSPHERIC METALS IN THE SURFACE OCEAN

During the spring in the Sargasso Sea, nutrients introduced during winter mixing are trapped within the sunlit layer as the surface ocean stratifies, providing conditions necessary for the spring bloom to occur. ${\text{NO}}_{3}^{-}$ availability is high in the spring relative to the rest of the year, and has been shown to drive the system toward Fe limitation (Moore et al., 2006). Moore et al. (2006) show that the supply of Fe from atmospheric deposition could influence the initiation, duration, and magnitude of the spring bloom by regulating phytoplankton ${\text{NO}}_{3}^{-}$ uptake, thereby stimulating new primary production. The results of the incubation experiments in this study support the hypothesis of Moore et al. (2006) regarding the importance of atmospheric deposition as a source of Fe that regulates the spring bloom, as the greatest increases in photosynthetic biomass were observed in treatments where Fe (and N) were available.

In addition to supplying Fe, our results also suggest an additional role for atmospheric deposition during the spring bloom. Atmospheric deposition can influence phytoplankton community composition during the spring bloom by providing other trace metal micronutrients, such as Co, that could limit growth as the bloom progresses. These findings are in agreement with those of Shelley et al. (2010), who observed that the nutrient-like profile for dissolved Co displayed a surface maximum following a period of strong aeolian deposition in the Sargasso Sea. The results presented here suggest that aerosol-derived Co is bioavailable and can support the growth of phytoplankton. Our results also show that in addition to Fe and Co, aerosols provide many biologically important trace metals needed for phytoplankton growth (**Figure 6**), and that these nutrients get consumed as cells grow (**Figure 7**). The selective enhancement of *Synechococcus* abundance in the oceanic experiment demonstrates the potential for these aerosol-derived metals to cause shifts in community composition by relieving the nutrient limitation of certain taxa and altering competition among different subpopulations.

The influence and fate of atmospheric metals may differ spatially in the Sargasso Sea. In the oceanic experiment, metals were consumed rapidly as phytoplankton grew. In contrast, little drawdown occurred following aerosol additions in the coastal experiment, suggesting that coastal phytoplankton assemblages may have been less stressed for these metals. This difference between sites may stem from a greater input of metals to coastal waters from terrigenous sources (e.g., runoff, groundwater) and resuspension of shelf sediments, compared to the open ocean that has fewer sources of trace metals. Alternately, the results could point toward a higher degree of ${\text{NO}}_{3}^{-}$ limitation in the coastal population, which may have precluded greater uptake of trace metal. Indeed, all of the ${\text{NO}}_{3}^{-}$ contributed by Aerosol 2 was consumed by the end of the coastal experiment, whereas >2µM residual ${\text{NO}}_{3}^{-}$ persisted in the oceanic experiment (**Figure 5**). If coastal phytoplankton became ${\text{NO}}_{3}^{-}$ limited during the experiment, this may have precluded efficient uptake of aerosol-derived trace metals.

The effect of atmospheric metal deposition on bloom development depends on the nutrient status of the resident phytoplankton assemblage, the aerosol depositional flux to the ocean’s surface, and the chemical composition of the aerosol. The Sargasso Sea receives a substantial input of Sahara dust during the summer months, whereas in the winter aerosols from North America predominate (Prospero et al., 1996). In this study both Aerosol samples contained a mixture of material from Sahara and North American sources (not shown); however, Aerosol 2 had more anthropogenic material than Aerosol 1 and caused a stronger growth response in the oceanic phytoplankton community that was commensurate with metal drawdown. The flux of soluble metal (i.e., the fraction that is likely available to phytoplankton) depends strongly on the relative proportions of anthropogenic versus mineral particles a region receives. Anthropogenic aerosols can be >10-fold enriched relative to average crustal abundances in metals such as Ni, Cu, Zn, Pb, and Cd (Chen et al., 2008; Chase et al., 2011). More variability exists in the total Fe content in atmospheric deposition with respect to anthropogenic and mineral sources; however, anthropogenic aerosols tend to have much higher fractional metal solubility compared to mineral aerosol (i.e., a greater proportion of the total Fe is water soluble, Sholkovitz et al., 2012). Similar solubility trends have been observed for Cu in the North Atlantic, where the fractional solubility of Cu in anthropogenic sources ranges from 10 to 100%, while Cu in mineral aerosol from the Sahara desert is less soluble (1–7%; Sholkovitz et al., 2010). Accordingly, the amount of soluble, and presumably bioavailable, metals arriving at a location may be strongly influenced by even small inputs of anthropogenic aerosol.

Since the spring bloom is a transitional time for air masses reaching the Sargasso Sea (Prospero et al., 1996), the amount of bioavailable metals delivered via atmospheric deposition would depend strongly on the relative proportion of anthropogenic aerosols in a given deposition event. In this study, Aerosol 2 was enriched in V relative to Al, a property that indicates greater anthropogenic input. The higher soluble metal content and greater fertilization potential of Aerosol 2 (**Figures 6** and **4**), may therefore be linked to an anthropogenic source. Our results suggest that the effect of atmospheric deposition during the spring bloom therefore depends not only on the amount of deposition, but the specific source and chemical composition of the aerosol deposited. The greater input of anthropogenic aerosols in the late winter/early spring as the bloom develops could be important in determining the magnitude of ${\text{NO}}_{3}^{-}$ drawdown, as has been proposed for aerosol Fe (Moore et al., 2006), as well as in shaping phytoplankton community composition, as observed for Aerosol 2 in this study. Coupled ecosystem-biogeochemical models that include atmospheric deposition should include this distinction where possible to explore its effect on productivity.

## CONCLUSION

In this study we sought to understand the effect of atmospheric metal deposition on oceanic and coastal Sargasso Sea phytoplankton communities. This study shows that aerosol deposition contributes biologically important constituents in addition to Fe and N to seawater, and that these bioavailable metals get consumed over time scales commensurate with cell growth. In particular, aerosol-derived micronutrients like Co, for which certain taxa have absolute requirements (Sunda and Huntsman, 1995a), may favor the growth of certain subpopulations and alter phytoplankton community composition. In contrast, during the spring season when these experiments were conducted, Cu toxicity did not appear to be an important factor influencing phytoplankton community composition.

The solubility of atmospheric metals is an important factor affecting metal bioavailability, and the relative proportions of anthropogenic and mineral sources in atmospheric deposition has the potential to influence phytoplankton growth and community composition in a given region. Accordingly, different types of aerosol can elicit very different growth responses from phytoplankton communities. These responses can differ between distinct populations despite their relatively close geographic proximity, and can reflect different antecedent conditions to which the cells were exposed with respect to metal availability.

## Conflict of Interest Statement

The authors declare that the research was conducted in the absence of any commercial or financial relationships that could be construed as a potential conflict of interest.
